# Characterization of Flavor Profile of “Nanx Wudl” Sour Meat Fermented from Goose and Pork Using Gas Chromatography–Ion Mobility Spectrometry (GC–IMS) Combined with Electronic Nose and Tongue

**DOI:** 10.3390/foods12112194

**Published:** 2023-05-30

**Authors:** Xin Zhao, Jianying Feng, Luca Laghi, Jing Deng, Xiaofang Dao, Junni Tang, Lili Ji, Chenglin Zhu, Gianfranco Picone

**Affiliations:** 1 College of Food Science and Technology, Southwest Minzu University, Chengdu 610041, China; zhaoxinhh@outlook.com (X.Z.); fengjianying@stu.swun.edu.cn (J.F.);; 2 Department of Agricultural and Food Sciences, University of Bologna, 47521 Cesena, Italy; 3 Cuisine Science Key Laboratory of Sichuan Province, Sichuan Tourism University, Chengdu 610100, China; 4 Meat Processing Key Lab of Sichuan Province, Chengdu University, Chengdu 610106, China

**Keywords:** fermented meat, volatile compounds, GC–IMS, intelligent sensory, chemometrics

## Abstract

Sour meat is a highly appreciated traditional fermented product, mainly from the Guizhou, Yunnan, and Hunan provinces. The flavor profiles of sour meat from goose and pork were evaluated using gas chromatography–ion mobility spectrometry (GC–IMS) combined with an electronic nose (E-nose) and tongue (E-tongue). A total of 94 volatile compounds were characterized in fermented sour meat from both pork and goose using GC–IMS. A data-mining protocol based on univariate and multivariate analyses revealed that the source of the raw meat plays a crucial role in the formation of flavor compounds during the fermentation process. In detail, sour meat from pork contained higher levels of hexyl acetate, sotolon, heptyl acetate, butyl propanoate, hexanal, and 2-acetylpyrrole than sour goose meat. In parallel, sour meat from goose showed higher levels of 4-methyl-3-penten-2-one, n-butyl lactate, 2-butanol, (E)-2-nonenal, and decalin than sour pork. In terms of the odor and taste response values obtained by the E-nose and E-tongue, a robust principal component model (RPCA) could effectively differentiate sour meat from the two sources. The present work could provide references to investigate the flavor profiles of traditional sour meat products fermented from different raw meats and offer opportunities for a rapid identification method based on flavor profiles.

## 1. Introduction

Sour meat is a traditional fermented product, mainly from the Guizhou, Yunnan, and Hunan provinces, where it is known as Nanx Wudl [1]. Its manufacture is usually carried out at an artisanal level based on non-standardized production protocols, where salt, rice, and seasonings, such as Chinese prickly ash and chili, are added to the meat. Fermentation is mainly carried out by taking advantage of naturally occurring lactic acid bacteria (LAB) and requires approximately 1–2 months under anaerobic conditions [2]. The meat most often used is pork, but others may be employed, such as the more expensive goose, which plays a special role from the consumer’s perspective because of its strong associations with the traditions of local Chinese communities [3]. From this point of view, it is worth noting that China accounts for as much as 70% of all the goose meat produced and consumed in the world [4].

Independent of the meat source in these fermented products, sour meat has attracted increasing attention outside the typical production areas— mainly due to its unique sensorial characteristics, but also due to its positive compositional traits, such as the richness in probiotics or the absence of nitrites. This brought about an increasing number of scientific works investigating various aspects of its production and characteristics. For instance, Lv et al. investigated the effect of fermentation temperature on the quality, bacterial community, and metabolites of sour meat. Their results showed that reduction in pH, thiobarbituric acid reactive substances (TBARS), and water content and an increase in lactic acid, free amino acids, and the number and amount of volatile compounds occurred as the fermentation temperature and time increased [5,6]. Lv et al. found that sour meat samples inoculated with *S. cerevisiae* LXPSC1 had better sensory characteristics than their naturally fermented counterparts, together with higher levels of pH, ethanol, free amino acids, and volatile organic compounds [7]. Zhang et al. found that the double-starter culture (*Lactobacillus curvatus* LAB26 and *Pediococcus pentosaceus* SWU73571) increased the L* and a* values, amino nitrogen content, and free amino acid content of sour meat significantly while also lowering the b* value; lowering the levels of nitrite, biogenic amines, total volatile basic nitrogen, and malondialdehyde; and restraining the coliform count [8]. Wang et al. found that low-salt fermentation can accelerate sourmeat maturation and facilitate the oxidation and decomposition of protein and fat and that it is more conducive to sour meat fermentation and to distinct fermented flavor production [9].

The main sensory characteristic that has been found to drive consumer preference and acceptance of fermented meat products is flavor [10]. Gas chromatography coupled with mass spectrometry (GC–MS) is considered the technique of choice for the qualitative and quantitative detection of volatile compounds in foods. In recent years, gas chromatography–ion mobility spectrometry (GC–IMS) has been increasingly used for flavor characterization in the food industry, because it can effectively distinguish the differences in flavor between products [11]. GC–IMS is an analytical technique that uses the difference in the migration rate of gas-phase ions in an electric field to characterize chemical substances [12]. It combines the excellent separation capacity of GC with the high sensitivity and fast response of IMS, granting a high accuracy of analysis [13]. To have a comprehensive view of the sensory characteristics of a food, it is ideal to couple this technique with an electronic nose and tongue. These devices are designed to mimic human olfactory and gustatory perception, respectively, without subjective judgements. They consist of a series of sensors designed to gain an overall fingerprint of the molecular profiles that give rise to complex odors and flavors [14]. Their application offers numerous advantages, among them rapidity of response, ease of use, reliability, and accuracy [15].

Despite the importance of aroma in determining consumer preferences for fermented meat foods and the potential of the E-nose, E-tongue, and GC–IMS for the purpose, few studies have evaluated their combination in this area. Moreover, most of the studies have considered products specifically manufactured at a laboratory scale or obtained at the retail level, which were industrially produced [16,17]. Finally, to the best of our knowledge, no studies have investigated the effects of different meat sources on the final product’s flavor profile, even though it has been demonstrated that the action of fermenting microorganisms (particularly LAB), which leads to the final flavor profile, is deeply influenced by the starting raw material [18,19].

To fill these numerous gaps, we attempted, for the first time, to discriminate traditional sour meat based on goose and pork by means of an E-nose and E-tongue, and to obtain their flavor features through a metabolomics approach based on GC–IMS. This work could provide a framework for investigating the flavor profiles of traditional sour meats fermented from pork and goose through GC–IMS and could offer opportunities for a rapid identification method based on overall flavor characteristics by means of an E-nose and E-tongue.

## 2. Materials and Methods

### 2.1. Experimental Design

In accordance with the traditional artisanal procedure for producing sour meat (Nanx Wudl) in the Yunnan province, fresh pork (Large White breed) and goose meat (Chinese Goose breed) were cut into small pieces (around 3 cm × 5 cm × 0.6 cm and 200 g each), and then mixed with 3% salt and pickled in a refrigerator at 4 °C for 24 h. Next, 1% pepper was added, along with 7% glutinous rice, fried to golden yellow and ground into coarse-grained, and 1% glutinous rice, fried to golden yellow and steamed. Finally, the ingredients were placed in ten sealed containers (five for pork and five for goose) and spontaneously fermented at room temperature (approximately 15 °C) for 60 days.

### 2.2. Electronic Nose Analysis

**Figure 1:**
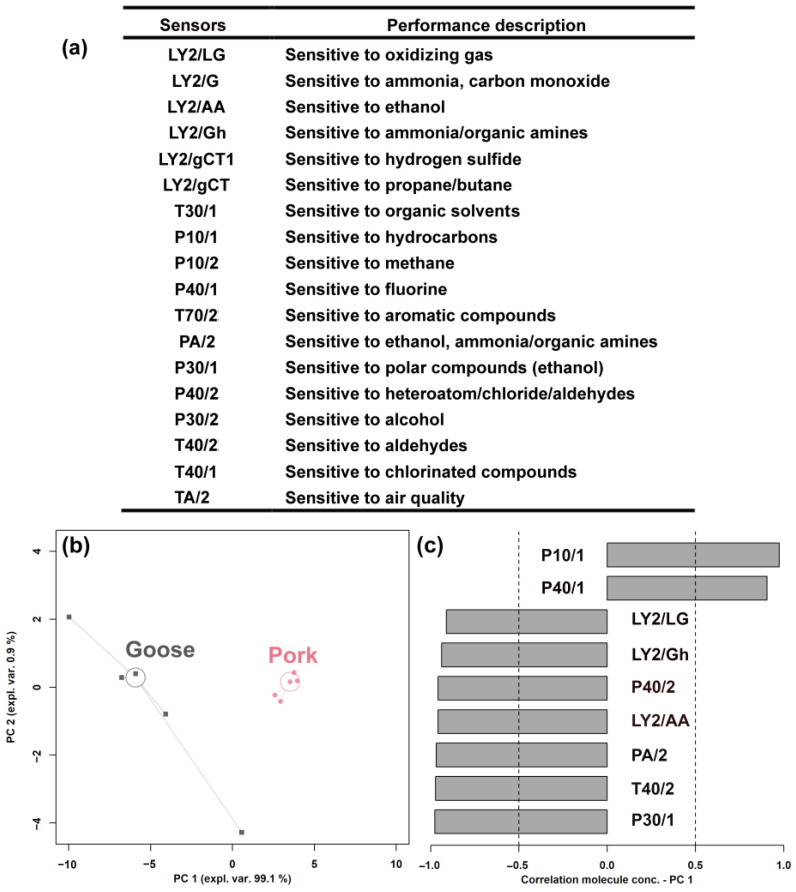
Performance description of the E-nose sensors (**a**); an RPCA model based on the E-nose response data, presented as a score plot (**b**); and a Pearson correlation plot of the loadings (**c**).

A commercial E-nose (FOX 4000, Alpha MOS, Toulouse, France), equipped with an injection system, 18 sensor chambers, a mass flow controller, and an acquisition board with a microcontroller, was used to discriminate different sour meat samples. The main pieces of information granted by each sensor are shown in Figure 1. In order to fulfill the requirement of E-nose analysis, 0.25 g of sour meat samples was put into a 10 mL headspace bottle, and then the samples were incubated at 70 °C for 5 min and manually injected. The measurement and rinsing phases took 120 s and 240 s, respectively. The observation of each sample was repeated five times, and three stable sets of data were retained. The average value for each sample was included in an RPCA plot.

### 2.3. Electronic Tongue Analysis

E-tongue analysis was performed by the α-ASTREE (equipped with sixteen autosampler carousel positions, Alpha MOS, Toulouse, France), which provided seven sensors for sourness (AHS), saltiness (CTS), umami (NMS), sweetness (ANS), bitterness (SCS), and two reference electrodes (PKS and CPS) [20,21,22]. Sour meat samples (20.0 g) were mixed with 200 mL of deionized water to extract the taste substances. After the mixed solution was centrifuged 2265× *g* for 10 min at 4 °C, the water phase (100 mL) was obtained for E-tongue analysis. Each collection time was set to 120 s. The stirring rate was set to 60 rpm, and the cleaning time was 30 s. Deionized water was used as a cleaning solution. The average value measured between 100 and 120 s was taken as the output value. Following the suggestion of Li et al. [20], each sample was repeated eight times, and the data of the last 5 stable sets were selected as the original data for analysis. The average value for each sample was included in an RPCA plot.

### 2.4. GC–IMS Analysis

The volatile compounds in the sour meat samples were analyzed using GC–IMS (Flavorspec, G.A.S. Instrument, Munich, Germany) with an MXT-WAX capillary column (30 m × 0.53 mm × 1 μm) (Restek, Mount Ayr, USA). Without any sample pre-treatment, 0.25 g of meat samples was accurately weighed and put into a 20 mL headspace (HS) vial with a magnetic screw seal cover. Then, the samples were incubated at 50 °C for 10 min. After incubation, 100 μL of the headspace sample was automatically injected into the injector (splitless mode) via a heated syringe at 65 °C. The column was kept at 60 °C, with the drift tube temperature at 45 °C. The drift gas flow was set to a constant flow rate of 150 mL/min. Nitrogen carrier gas (99.999% purity) was used, and the GC column flow rate was programmed as follows: 2 mL/min for 5 min, 10 mL/min for 10 min, 15 mL/min for 5 min, 50 mL/min for 10 min, and 100 mL/min for 10 min. Following the suggestions of previous papers [23,24], the retention index (RI) of volatile compounds was calculated using n-ketones C4–C9 as external references. Volatile compounds were identified by comparing their RI and ions’ drift time— that is, their migration time from the ionization source to the detector in the IMS chamber— with those of the standards in the GC–IMS library. In accordance with Guo et al. [25], the relevant calculation formula is as follows:

$$RI(x)=RI(x-1)+\frac{\left[RI(x+1)-RI(x-1)]\times [RT(x)-RT(x-1)\right]}{RT(x+1)-RT(x-1)}$$

*RT*(*x*): The retention time of the substance/min;
*RT*(*n*): The retention time of the n-ketones/min;
*n*: The number of carbon atoms in the n-ketones;
*x*: target to be carried out via qualitative and quantitative analysis;
*x* − 1: The component peaking before target x;
*x* + 1: The component peaking after target x.

Each sample was detected once, and the quantification of volatile compounds was based on the peak signal intensity. Using the Laboratory Analytical Viewer, Reporter, and Gallery Plot supported by the GC–IMS instrument, three-dimensional (3D) and two-dimensional (2D) topographic plots and gallery plots of the volatile compounds were constructed.

### 2.5. Statistical Analysis

Statistical analysis was performed in R computational language. Prior to the univariate analyses, the distribution of the data was brought to normality according to Box and Cox [26]. We used *t*-tests to look for significant differences between groups (*p* < 0.05).

Following the suggestions of previous studies [27,28], with the aim of obtaining an overall view of the data, robust principal component analysis (RPCA) models were set up based on the average values of the E-nose and E-tongue sensors and the molecules’ peak signal intensities, respectively. For each RPCA model, a score plot and a Pearson correlation plot of the loadings were calculated, to highlight the structure of the data and to find out the relationships between variables and the model components.

## 3. Results

### 3.1. Electronic Nose Analysis

The E-nose analyzer, equipped with 18 sensors, was used to identify different sour meat formulations and to assess their comprehensive flavor characteristics. The main performance of the sensors is described in Figure 1a, as suggested by Wen et al. [29]. The response from nine of the sensors was found to be significantly different between the two groups (*p* < 0.05). To obtain an overview of the trends of these sensors, their response values were employed as a basis for an RPCA model, shown in Figure 1b,c.

Sour meat samples fermented from goose and pork could be clearly distinguished when observed along PC 1, with goose mainly characterized by higher response values from the sensors LY/LG, LY/Gh, P40/2, LY2/AA, PA/2, T40/2, and P30/1 and by lower response values from the sensors P10/1 and P40/1.

### 3.2. Electronic Tongue Analysis

**Figure 2:**
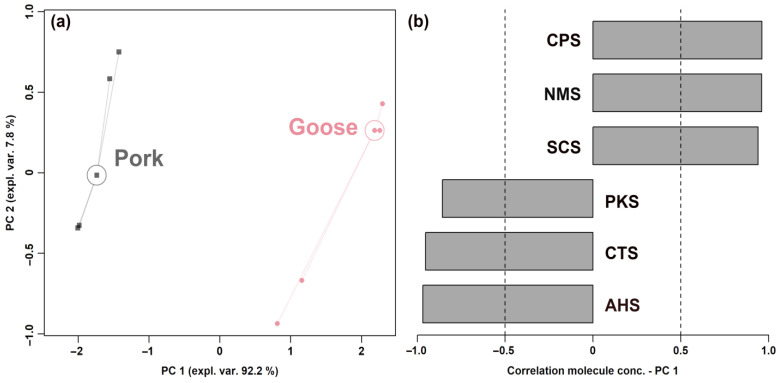
Score plot (**a**) and Pearson correlation plot (**b**) of the loadings of an RPCA model based on E-tongue response data.

The E-tongue analyzer, equipped with seven sensors, was used to identify different sour meat formulations and to assess their comprehensive taste characteristics. Six of the sensors gave a significantly different response when analyzing the two groups (*p* < 0.05). In order to obtain an overview of the trends of these sensors, their response values were employed as a basis for an RPCA model, shown in Figure 2.

The results showed that sour meat samples fermented from goose and pork were well distinguished along PC 1. Sour meat fermented from goose was mainly characterized by higher response values from the sensors CPS, NMS, and SCS and by lower response values from the sensors PKS, CTS, and AHS.

### 3.3. GC–IMS Analysis

**Figure 3:**
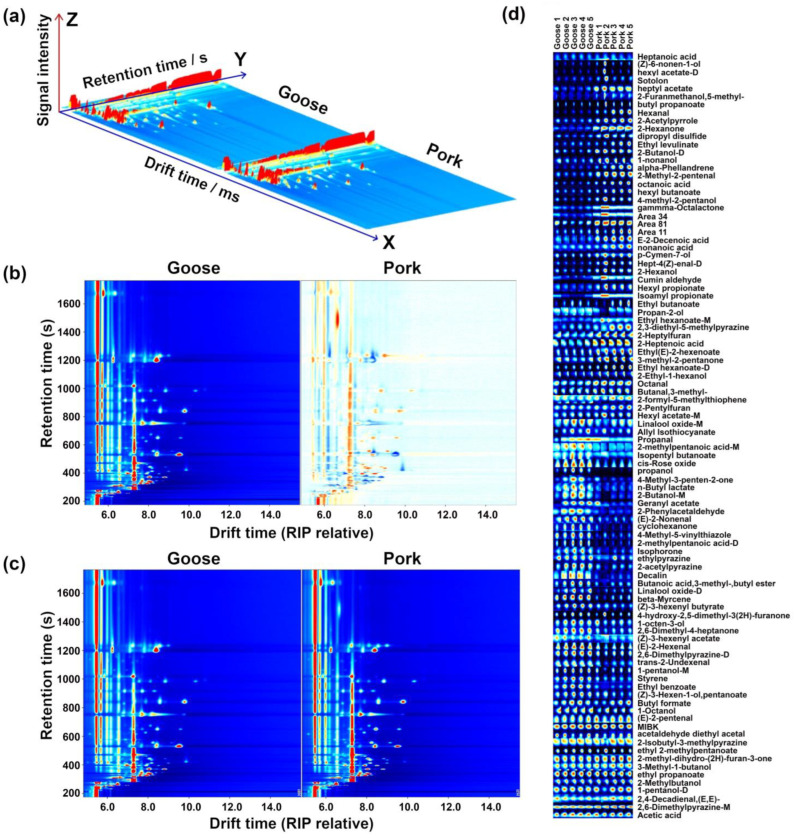
GC–IMS observations of sour meat fermented from goose and pork. (**a**) Their three-dimensional representation. (**b**) Their bird’s-eye view representation, with spectra from goose meat employed as a reference and the corresponding spectra from pork represented as differences from goose meat. In the latter case, red and blue highlight components that were over- and under-expressed, respectively. (**c**) Their representation as ion migration spectra, where the ions are numbered and then listed in (**d**) as gallery plots, in which the color was brighter, the content was higher.

The processing pipeline of the GC–IMS information about the volatile components in the samples from goose and pork meat is summarized by Figure 3.

The 3D representation of Figure 3a offers an unbiased visual impression that the samples from the two meat sources differed along large portions of the GC–IMS spectrum. This allows us to establish that GC–IMS is a technique well suited for distinguishing fermented meat from the two studied sources. The point-by-point differences between the two sets of samples in Figure 3b allow us to appreciate in finer detail that most of the peculiarities regarded compounds with retention times between 200 and 1000 s, whose ions showed drift times between 6.0 and 10.0 ms. A total of 94 compounds were identified, including ketones (**10**), acids (**8**), alcohols (**19**), aldehydes (**15**), esters (**27**), and others (**10**). The relevant information about each of them is provided in Table 1.

The topographic plots of Figure 3d allow us to visually appreciate the trends distinguishing the two sets of samples, and they demonstrate that many of the volatile compounds distinguishing fermented goose meat from fermented pork were acids and alcohols. For example, hexyl acetate, sotolon, heptyl acetate, butyl propanoate, hexanal, and 2-acetylpyrrole appeared as more concentrated in sour meat from fermented pork, while levels of 4-methyl-3-penten-2-one, n-butyl lactate, 2-butanol, (E)-2-nonenal, and decalin were higher in the samples from goose meat.

**Figure 4:**
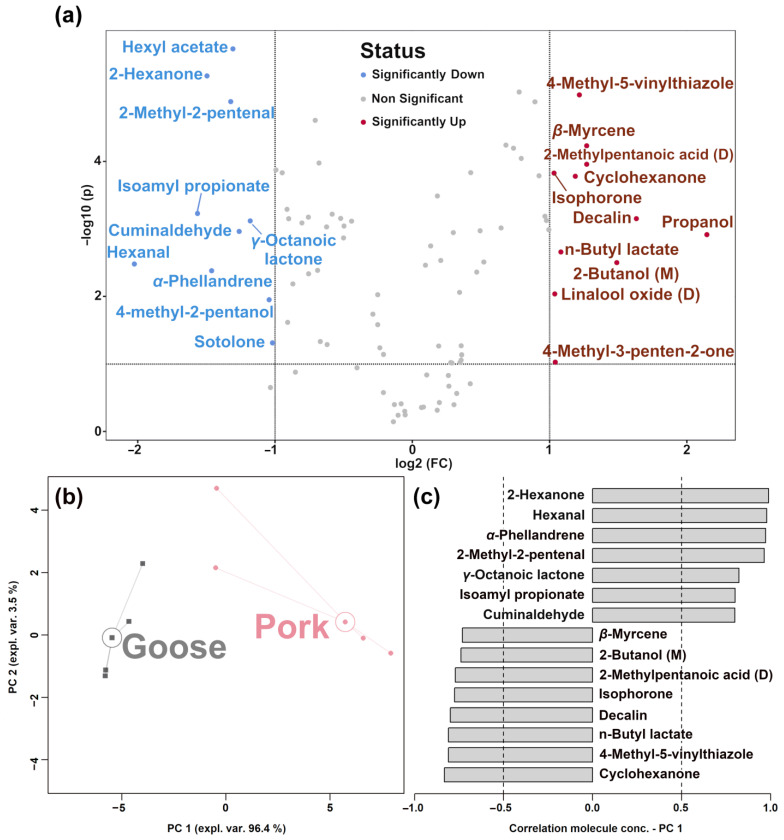
Volcano plot (**a**) indicating the changes in the concentrations of metabolites in sour meat samples from the two groups. The RPCA model was set up on the basis of the molecules selected by the volcano plot. In the score plot (**b**), squares and circles indicate goose and pork samples, respectively. The median of each sample group is indicated by wide, empty circles. The Pearson correlation plot of the loadings (**c**) shows the molecules with significant correlations between concentration and importance over PC 1 (*p* < 0.05).

In accordance with Zhu et al., to identify the molecules showing the highest differences between the two types of samples, a volcano plot was set up, which nicely combines the results of the *t*-test and fold-change analysis on a molecule-by-molecule basis [30]. Significantly different molecules with a fold change higher than 2 are shown in Figure 4a. To obtain an overview of the trends of these molecules, their signal intensities were employed as a basis for an RPCA model, as shown in Figure 4b,c.

In the score plot of Figure 4b, the PC 1 accounted for as much as 96.4% of the samples’ overall variability and perfectly summarized the differences between goose and pork samples, with negative and positive PC 1 scores, respectively. The Pearson correlation plot of the loadings of Figure 4c shows that sour meat fermented from goose has higher amounts of *β*-myrcene, 2-butanol, 2-methylpentanoic acid, isophorone, decaline, n-butyl lactate, 4-methyl-5-vinylthiazole, and cyclohexanone and lower concentrations of 2-hexanone, hexanal, *α*-phellandrene, 2-methyl-2-pentenal, *γ*-octanoic lactone, isoamyl propionate, and cuminaldehyde.

### 3.4. Correlation between E-Nose and GC–IMS

**Figure 5:**
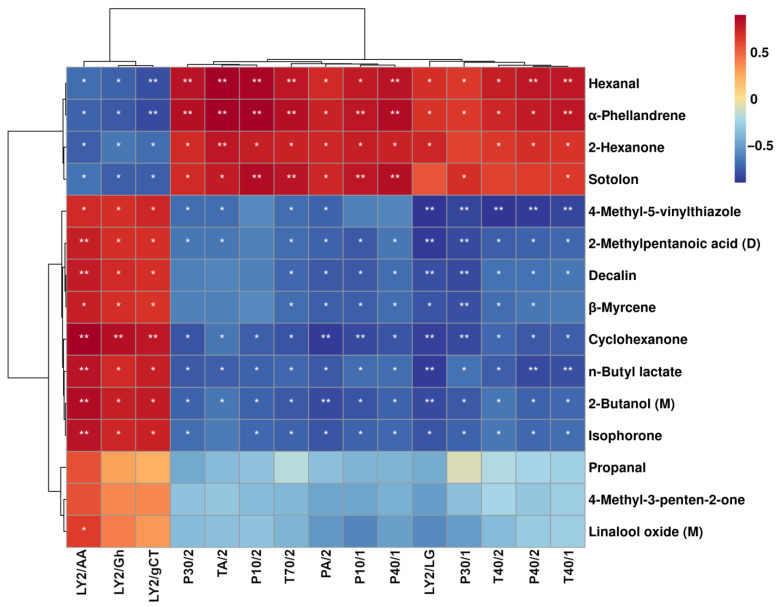
Spearman’s correlation heatmap showing the correlation between the significantly altered volatile compound levels and the electronic nose sensor responses. Colors represent correlation coefficients, with red and blue indicating positive and negative correlation, respectively. Asterisks * and ** stand for significance at *p* < 0.05 and *p* < 0.01, respectively.

The E-nose and GC–IMS can classify sour meat fermented from pork and goose meat from different points of view. For example, E-nose was able to provide overall information on the volatile compounds in each sample. In contrast, GC–IMS could provide the specific volatile profile of each sour meat. Therefore, in order to promote the overall performance of both techniques, the potential correlation between E-nose sensor responses and volatile compound levels detected by GC–IMS was analyzed, as shown in Figure 5.

As shown in Figure 5, the LY2/AA and LY2/Gh sensors were found to positively correlate with hexanal, *α*-phellandrene, and 2-hexanone. These compounds were characterized by GC–IMS at high levels in the sour meat fermented from pork. In contrast, the PA/2, P10/1, P40/1, LY2/LG, P30/1, T40/2, and P40/2 sensors were found to positively correlate with 2-methylpentanoic acid (D), decaline, *β*-myrcene, cyclohexanone, n-butyl lactate, 2-butanol (M), and isophorone. These compounds were characterized by GC–IMS at high levels in the sour meat fermented from goose meat. These results indicate that the E-nose sensor response values and volatile compound quantifications characterized through GC–IMS can discriminate the unique flavors of sour meat fermented from pork versus goose meat.

## 4. Discussion

Sour meat (Nanx Wudl) is a fermented meat product traditionally manufactured by minorities (the Dong, the Miao, the Dai, the Tujia, the Maonan, etc.) but increasingly appreciated in wider parts of China due to its unique flavor, richness in nutrients, and long shelf life [2,16,31]. However, studies evaluating the effects of different raw meats on the formation of the typical complex flavor during fermentation are still lacking. To shed light on the issue, the present study attempted, for the first time, to comprehensively characterize the flavor profiles of sour meat from goose and pork by means of GC–IMS combined with an electronic nose and tongue—a perfect combination for the purpose, but still rarely employed.

Nine of the eighteen sensors on the E-nose analyzer showed a significantly different response between the samples from goose meat and pork, evidencing that this tool is extremely sensitive to peculiarities in the overall flavor profiles connected to the raw materials for fermented meat. From this point of view, it is worth underlining that this tool is not able to identify the specific volatile compounds giving rise to the overall response; a specific high-throughput technique tailored for the purpose, such as GC–IMS, should be used in parallel to obtain fine-grained information, at least in the first stages of investigation. Six of the seven sensors on the E-tongue showed a significantly different response to the two tested products, demonstrating that, in this context, this technique gives interesting complimentary information from the point of view of taste attributes.

A total of 94 compounds was characterized in each of the tested samples by GC–IMS, by comparing their RI and ion drift time to the standards in the GC–IMS library and references [32,33,34,35], pertaining to ketones (**10**), acids (**8**), alcohols (**19**), aldehydes (**15**), esters (**27**), and others (**10**). Among them, twenty-one compounds exhibited significant differences between the two types of samples, namely hexyl acetate, 2-hexanone, 2-methyl-2-pentenal, isoamyl propionate, cuminaldehyde, hexanal, *γ*-octanoic lactone, *α*-phellandrene, 4-methyl-2-pentanol, sotolon, 4-methyl-5-vinylthiazole, *β*-myrcene, 2-methylpentanoic acid (D), cyclohexanone, isophorone, decaline, propanol, n-butyl lactate, 2-butanol (M), linalool oxide (D), and 4-methyl-3-penten-2-one.

2-Butanol is a flavor-enriching substance with a sweet and pleasant scent [36]. The compound is derived from the reduction of 2-butanone. Moreover, it can also derive from pyruvate, similar to 1-propanol [37]. In turn, pyruvate generation could be attributed to amino acid metabolism, active in lactic acid bacteria, particularly for aspartate [38]. The distinct content of 2-butanol in sour meat fermented from goose meat and pork could be explained, at least in part, by the different amino acid profiles of goose meat versus pork [39,40].

Hexanal and 2-methyl-2-pentenal, belonging to the chemical class of aldehydes, have been found in beef meat [41] and chicken meat [42] and are considered to be useful markers of lipid oxidation [43,44]. Choi et al. found, by studying plant substrates during drying, that hexanal and 2-methyl-2-pentenal are produced from the action of residual enzymes so that, especially at low drying temperatures, their concentration is proportional to the residual humidity of the sample [45]. From these studies, it could be inferred that the different contents of hexanal and 2-methyl-2-pentenal in sour meat fermented from goose meat versus pork could be linked to different activity of residual enzymes, in turn leading to variable extents of lipid oxidation in the final product [46].

2-Hexanone, like many ketones, has an unpleasant, pungent odor [47]. By observing silver carp during chill storage, Jia et al. found that 2-hexanone was initially absent and increased gradually with storage time, showing a good correlation with microbial growth [48]. Similar to 2-hexanone, 2-methyl-2-pentenal, 2-methylpentanoic acid, and 4-methyl-3-penten-2-one were found to be linked to microbiota metabolism, though they were mainly linked to lipid oxidation, carbohydrate fermentation, and amino acid degradation [49,50].

Hexyl acetate is an ester with a pleasant fruity scent, which can usually be found in meat products. Li et al. found that hexanal was first reduced to 1-hexanol by *Lb. fermentum* and subsequently converted into hexyl acetate by *P. kluyveri* during pork fermentation [51]. Similarly, Jiang et al. found that there were positive correlations between the levels of *Leuconostoc* and *Lactobacillus* versus hexyl acetate in smoked horse meat sausages [52]. Zhang et al. found that hexyl acetate was a crucial positive contributor to the flavor profile of unsmoked bacon [53].

Overall, the trends we observed of the above-mentioned molecules seem to confirm that microorganisms’ role in determining flavor profile is determined by the raw materials, particularly the meat. The trends of other molecules seem to confirm this observation. Linalool oxide can contribute to woody and floral aromas [54]. Sotolon is formed through the aldol condensation of α-ketobutyric acid, produced by threonine and acetaldehyde, which in turn result from the oxidation of ethanol generated, by the glucose metabolism of yeasts during fermentation, among other processes. Confirming this, Ohata et al. used 10% commercial koji and 10% salts to ferment a pork meat sauce for 12 months, and they found that the main odor contributors in their fermented meat sauce were sotolon and ethyl furaneol, which gave the meat sauce a sweet and caramel-like note [55]. Other compounds such as *α*-phellandrene, *β*-myrcene, and cuminaldehyde can be considered plant-derived [56,57,58]. The presence of these compounds in the final product is most likely due to the spices added to fresh meat, particularly pepper.

In the present study, it is worth noting that the combination of the E-nose, E-tongue, and GC–IMS could improve the overall performance of all techniques and provide a comprehensive characterization of sour meat fermented from pork and goose meat. In particular, the correlation between E-nose response values and GC–IMS molecule peak areas highlighted that lipid-oxidation-related compounds (such as hexanal and 2-hexanone) played important roles in discriminating sour meat fermented from goose meat and from pork. Moreover, a few of the E-nose sensors (such as PA/2, P10/1, P40/1, LY2/LG, T40/2, and P40/2), which exhibited significantly higher response values for the above compounds, could be considered potential candidates for developing targeted analysis methods by means of an E-nose for practical sample analysis.

## 5. Conclusions

In this study, for the first time, the flavor features of sour meat traditionally fermented from goose and pork were systematically characterized by means of GC–IMS, an E-nose, and an E-tongue. Taking advantage of a protocol based on univariate and multivariate analyses, we found that the raw material played a crucial role in introducing peculiarities to large portions of the flavor profile, so that sour meat from goose was readily distinguishable from that based on pork. Though the E-nose and E-tongue are able to grant an overall view of the odor and taste features of samples, it is still necessary to apply high-throughput techniques in parallel, to spot the specific volatile compounds conveying the overall response. Notably, none of the tested analyses required sample preparation, which implies that these operations are simple, fast, and nondestructive. Therefore, the present work could provide a basis for investigating the flavor profiles of traditional sour meats fermented from different meat sources and shed light on establishing more comprehensive and rapid methods for identifying flavor characteristics.

## Figures and Tables

**Table 1:** Molecules’ peak areas (mean ± sd) characterized by GC–IMS in sour meat fermented from both goose and pork.

Count	Compound	CAS	Molecule Formula	MW *	RI	RT (s)	DT (ms)	Goose	Pork	*p*-Value
Ketones	2,6-Dimethyl-4-heptanone	C108838	C_9_H_18_O	142.2	1210.4	756.074	1.77916	1.15 × 10^3^ ± 43.80	7.19 × 10^2^ ± 1.13 × 10^2^	0.008
	2-Hexanone	C591786	C_6_H_12_O	100.2	1093.1	436.042	1.49671	7.90 × 10^2^ ± 66.90	2.23 × 10^3^ ± 2.82 × 10^2^	0.000
	4-Hydroxy-2,5-dimethyl-3(2 H)-furanone	C3658773	C_6_H_8_O_3_	128.1	1053.3	371.81	1.61084	3.05 × 10^3^ ± 3.90 × 10^2^	2.29 × 10^3^ ± 1.15 × 10^3^	0.139
	4-Methyl-3-penten-2-one	C141797	C_6_H_10_O	98.1	1106.1	462.228	1.44482	7.86 × 10^2^ ± 4.19 × 10^2^	3.88 × 10^2^ ± 1.79 × 10^2^	0.093
	Isophorone	C78591	C_9_H_14_O	138.2	1115	480.134	1.25323	1.01 × 10^3^ ± 1.40 × 10^2^	4.97 × 10^2^ ± 63.20	0.000
	2-Methyl-dihydro-(2 H)-Furan-3-one	C3188009	C_5_H_8_O_2_	100.1	1280.5	1019.37	1.06662	1.74 × 10^3^ ± 2.62 × 10^2^	2.07 × 10^3^ ± 93.20	0.056
	MIBK	C108101	C_6_H_12_O	100.2	1021.3	338.292	1.47542	2.36 × 10^2^ ± 19.10	3.54 × 10^2^ ± 50.70	0.001
	3-Methyl-2-pentanone	C565617	C_6_H_12_O	100.2	1056.3	374.988	1.47533	3.54 × 10^3^ ± 7.26 × 10^2^	4.16 × 10^3^ ± 2.62 × 10^2^	0.155
	2-Acetylpyrrole	C1072839	C_6_H_7_NO	109.1	1082.5	414.813	1.49823	8.98 × 10^2^ ± 2.03 × 10^2^	1.78 × 10^3^ ± 2.21 × 10^2^	0.003
	2-Acetylpyrazine	C22047252	C_6_H_6_N_2_O	122.1	1017.1	333.894	1.20618	8.62 × 10^2^ ± 1.40 × 10^2^	4.40 × 10^2^ ± 85.10	0.004
Acids	Acetic acid	C64197	C_2_H_4_O_2_	60.1	1447.3	1673.557	1.05277	1.73 × 10^4^ ± 1.07 × 10^3^	1.52 × 10^4^ ± 1.77 × 10^3^	0.101
	Octanoic acid	C124072	C_8_H_16_O_2_	144.2	1174.4	628.473	1.44089	9.98 × 10^2^ ± 1.52 × 10^2^	1.87 × 10^3^ ± 3.04 × 10^2^	0.002
	2-Methylpentanoic acid (D)	C97610	C_6_H_12_O_2_	116.2	1029.9	347.315	1.59258	2.77 × 10^2^ ± 37.50	1.15 × 10^2^ ± 19.90	0.000
	2-Methylpentanoic acid (M)	C97610	C_6_H_12_O_2_	116.2	1028.9	346.226	1.26389	4.00 × 10^2^ ± 46.80	2.57 × 10^2^ ± 24.30	0.001
	Heptanoic acid	C111148	C_7_H_14_O_2_	130.2	1082.4	414.564	1.3611	3.66 × 10^2^ ± 12.40	4.37 × 10^2^ ± 54.40	0.013
	Nonanoic acid	C112050	C_9_H_18_O_2_	158.2	1279.5	1015.116	1.54967	1.50 × 10^3^ ± 2.57 × 10^2^	2.40 × 10^3^ ± 1.65 × 10^2^	0.010
	E-2-Decenoic acid	C334496	C_10_H_18_O_2_	170.3	1334.9	1232.645	1.48495	2.12 × 10^2^ ± 40.80	3.27 × 10^2^ ± 97.00	0.054
	2-Heptenoic acid	C18999285	C_7_H_12_O_2_	128.2	1207	744.053	1.40605	1.26 × 10^3^ ± 2.56 × 10^2^	3.10 × 10^3^ ± 2.42 × 10^2^	0.001
Alcohols	1-Octen-3-ol	C3391864	C_8_H_16_O	128.2	1447.5	1674.263	1.1622	4.63 × 10^3^ ± 6.10 × 10^2^	3.62 × 10^3^ ± 9.00 × 10^2^	0.096
	1-Pentanol (D)	C71410	C_5_H_12_O	88.1	1253.8	914.653	1.51902	1.83 × 10^3^ ± 1.34 × 10^2^	1.52 × 10^3^ ± 4.16 × 10^2^	0.257
	1-Pentanol (M)	C71410	C_5_H_12_O	88.1	1254.1	915.518	1.25162	1.61 × 10^3^ ± 1.27 × 10^2^	1.33 × 10^3^ ± 3.01 × 10^2^	0.199
	2-Hexanol	C626937	C_6_H_14_O	102.2	1235.1	843.655	1.56671	4.13 × 10^3^ ± 1.04 × 10^3^	7.17 × 10^3^ ± 9.86 × 10^2^	0.016
	3-Methyl-1-butanol	C123513	C_5_H_12_O	88.1	1207.6	746.268	1.24551	1.15 × 10^3^ ± 40.60	1.07 × 10^3^ ± 1.03 × 10^2^	0.235
	2-Methylbutanol	C137326	C_5_H_12_O	88.1	1214.5	770.435	1.24551	2.27 × 10^3^ ± 1.71 × 10^2^	1.97 × 10^3^ ± 1.23 × 10^2^	0.036
	p-Cymen-7-ol	C536607	C_10_H_14_O	150.2	1281.2	1021.821	1.32996	5.51 × 10^3^ ± 1.15 × 10^3^	1.03 × 10^4^ ± 1.82 × 10^3^	0.006
	(Z)-6-nonen-1-ol	C35854865	C_9_H_18_O	142.2	1181.1	652.231	1.75376	8.75 × 10^2^ ± 30.50	1.59 × 10^3^ ± 9.61 × 10^2^	0.016
	4-Methyl-2-pentanol	C108112	C_6_H_14_O	102.2	1180.5	650.067	1.54657	2.02 × 10^3^ ± 5.14 × 10^2^	4.15 × 10^3^ ± 1.43 × 10^3^	0.009
	1-Nonanol	C143088	C_9_H_20_O	144.3	1144.7	540.011	1.53779	2.82 × 10^3^ ± 8.05 × 10^2^	3.71 × 10^3^ ± 9.29 × 10^2^	0.105
Alcohols	Linalool oxide (D)	C60047178	C_10_H_18_O_2_	170.3	1087.7	425.155	1.80714	2.61 × 10^3^ ± 7.70 × 10^2^	1.27 × 10^3^ ± 3.23 × 10^2^	0.016
	Linalool oxide (M)	C60047178	C_10_H_18_O_2_	170.3	1082.4	414.503	1.25444	4.16 × 10^2^ ± 64.60	3.2 × 610^2^ ± 68.00	0.137
	2-Ethyl-1-hexanol	C104767	C_8_H_18_O	130.2	1030.4	347.834	1.4048	3.83 × 10^3^ ± 6.13 × 10^2^	4.42 × 10^3^ ± 4.86 × 10^2^	0.129
	2-Butanol (D)	C78922	C_4_H_10_O	74.1	1016.3	333.005	1.33429	1.41 × 10^3^ ± 3.90 × 10^2^	2.71 × 10^3^ ± 3.06 × 10^2^	0.010
	2-Butanol (M)	C78922	C_4_H_10_O	74.1	1016	332.708	1.14461	6.46 × 10^2^ ± 1.90 × 10^2^	2.32 × 10^2^ ± 85.80	0.007
	Propanol	C71238	C_3_H_8_O	60.1	976.8	295.222	1.24209	6.75 × 10^2^ ± 2.14 × 10^2^	1.54 × 10^2^ ± 37.60	0.000
	Propan-2-ol	C67630	C_3_H_8_O	60.1	940.1	275.184	1.08085	7.06 × 10^2^ ± 60.40	5.53 × 10^2^ ± 1.74 × 10^2^	0.207
	1-Octanol	C111875	C_8_H_18_O	130.2	1030.2	347.582	1.46611	7.99 × 10^3^ ± 3.00 × 10^2^	7.04 × 10^3^ ± 4.65 × 10^2^	0.037
	2-Furanmethanol, 5-methyl-	C3857258	C_6_H_8_O_2_	112.1	949.8	280.488	1.26173	1.93 × 10^2^ ± 46.40	9.20 × 10^1^ ± 9.12	0.001
Aldehydes	Cumin aldehyde	C122032	C_10_H_12_O	148.2	1240.6	862.903	1.33504	3.60 × 10^3^ ± 1.00 × 10^3^	8.59 × 10^3^ ± 2.16 × 10^3^	0.005
	*trans*-2-Undecenal	C53448070	C_11_H_20_O	168.3	1335.1	1233.494	1.56546	3.86 × 10^3^ ± 2.27 × 10^2^	2.22 × 10^3^ ± 5.03 × 10^2^	0.017
	2,4-Decadienal,(E,E)-	C25152845	C_10_H_16_O	152.2	1315.6	1156.984	1.41449	3.79 × 10^2^ ± 16.70	4.62 × 10^2^ ± 51.90	0.005
	(Z)-4-Heptenal	C6728310	C_7_H_12_O	112.2	1272	985.747	1.62114	2.59 × 10^3^ ± 4.75 × 10^2^	4.85 × 10^3^ ± 1.84 × 10^3^	0.010
	2-Hexenal	C6728263	C_6_H_10_O	98.1	1210.7	757.253	1.50243	1.28 × 10^4^ ± 8.16 × 10^2^	7.70 × 10^3^ ± 1.07 × 10^3^	0.006
	2-Methyl-2-pentenal	C623369	C_6_H_10_O	98.1	1153.1	556.885	1.5028	1.03 × 10^3^ ± 2.27 × 10^2^	2.55 × 10^3^ ± 2.73 × 10^2^	0.001
	Hexanal	C66251	C_6_H_12_O	100.2	1073.1	395.761	1.55606	5.58 × 10^2^ ± 89.50	2.27 × 10^3^ ± 9.09 × 10^2^	0.001
	(E)-2-Nonenal	C18829566	C_9_H_16_O	140.2	1144.7	540.011	1.40541	1.37 × 10^3^ ± 1.13 × 10^2^	7.37 × 10^2^ ± 58.30	0.000
	(E)-2-Pentenal	C1576870	C_5_H_8_O	84.1	1094.4	438.764	1.36432	1.01 × 10^3^ ± 73.90	1.05 × 10^3^ ± 1.34 × 10^2^	0.975
	2-Formyl-5-methylthiophene	C13679704	C_6_H_6_OS	126.2	1108.5	467.078	1.17289	3.40 × 10^2^ ± 46.20	3.20 × 10^2^ ± 26.50	0.838
	Octanal	C124130	C_8_H_16_O	128.2	1006.9	323.217	1.39288	1.13 × 10^3^ ± 2.03 × 10^2^	1.54 × 10^3^ ± 75.20	0.033
	Butanal, 3-methyl-	C590863	C_5_H_10_O	86.1	909.8	258.669	1.19285	4.51 × 10^2^ ± 38.00	5.86 × 10^2^ ± 52.20	0.008
	Acetaldehyde diethyl acetal	C105577	C_6_H_14_O_2_	118.2	863.4	233.347	1.13106	5.32 × 10^2^ ± 90.20	4.71 × 10^2^ ± 21.70	0.563
	Propanal	C123386	C_3_H_6_O	58.1	842	221.677	1.03933	5.49 × 10^2^ ± 1.50 × 10^2^	4.89 × 10^2^ ± 1.33 × 10^2^	0.863
	2-Phenylacetaldehyde	C122781	C_8_H_8_O	120.2	1013.7	330.292	1.25765	1.95 × 10^3^ ± 3.40 × 10^2^	1.87 × 10^3^ ± 56.70	0.710
Esters	Isoamyl propionate	C105680	C_8_H_16_O_2_	144.2	1190.1	683.977	1.33833	5.35 × 10^3^ ± 2.37 × 10^3^	1.57 × 10^4^ ± 3.81 × 10^3^	0.005
	Ethyl-(E)-2-hexenoate	C27829727	C_8_H_14_O_2_	142.2	1334.8	1232.212	1.32568	3.78 × 10^3^ ± 2.68 × 10^2^	6.16 × 10^3^ ± 5.68 × 10^2^	0.001
	Hexyl propionate	C2445763	C_9_H_18_O_2_	158.2	1329.3	1210.43	1.43	4.53 × 10^3^ ± 6.92 × 10^2^	6.47 × 10^3^ ± 6.47 × 10^2^	0.021
	(Z)-3-hexenyl acetate	C3681718	C_8_H_14_O_2_	142.2	1315.4	1155.965	1.30104	3.60 × 10^2^ ± 23.20	2.92 × 10^2^ ± 23.80	0.015
	Hexyl acetate (D)	C142927	C_8_H_16_O_2_	144.2	1271.7	984.728	1.88721	1.25 × 10^3^ ± 91.60	2.58 × 10^3^ ± 2.26 × 10^3^	0.044
	(Z)-3-Hexen-1-ol, Pentanoate	C35852461	C_11_H_20_O_2_	184.3	1272.2	986.766	1.48608	1.53 × 10^3^ ± 28.50	1.26 × 10^3^ ± 3.50 × 10^2^	0.122
	Ethyl hexanoate (D)	C123660	C_8_H_16_O_2_	144.2	1234.6	841.923	1.79323	6.16 × 10^3^ ± 3.86 × 10^2^	9.92 × 10^3^ ± 1.89 × 10^3^	0.002
	Hexyl acetate (M)	C142927	C_8_H_16_O_2_	144.2	1271.9	985.651	1.38787	1.63 × 10^3^ ± 85.90	1.89 × 10^3^ ± 4.85 × 10^2^	0.383
Eesters	Ethyl benzoate	C93890	C_9_H_10_O_2_	150.2	1160.1	577.835	1.26386	9.73 × 10^2^ ± 1.61 × 10^2^	5.00 × 10^2^ ± 68.00	0.001
	γ-Octalactone	C104507	C_8_H_14_O_2_	142.2	1255.6	921.364	1.33563	4.68 × 10^3^ ± 9.39 × 10^2^	1.06 × 10^4^ ± 2.47 × 10^3^	0.002
	Ethyl hexanoate (M)	C123660	C_8_H_16_O_2_	144.2	1234.7	842.104	1.34131	3.84 × 10^3^ ± 4.45 × 10^2^	6.48 × 10^3^ ± 1.12 × 10^3^	0.002
	Hexyl butanoate	C2639636	C_10_H_20_O_2_	172.3	1188.4	678.191	1.48375	9.06 × 10^2^ ± 87.10	1.39 × 10^3^ ± 1.86 × 10^2^	0.003
	(Z)-3-hexenyl butyrate	C16491364	C_10_H_18_O_2_	170.3	1181.4	653.414	1.41322	9.61 × 10^2^ ± 58.10	7.61 × 10^2^ ± 1.07 × 10^2^	0.038
	Ethyl 2-methylpentanoate	C39255328	C_8_H_16_O_2_	144.2	1141.7	534.023	1.74779	1.12 × 10^4^ ± 1.16 × 10^2^	9.35 × 10^3^ ± 3.20 × 10^3^	0.137
	Sotolon	C28664359	C_6_H_8_O_3_	128.1	1097.4	444.752	1.61692	2.79 × 10^2^ ± 24.50	5.70 × 10^2^ ± 2.84 × 10^2^	0.007
	Ethyl levulinate	C539888	C_7_H_12_O_3_	144.2	1082	413.724	1.64736	4.97 × 10^2^ ± 55.40	7.92 × 10^2^ ± 2.66×10^2^	0.032
	Butanoic acid, 3-methyl-, Butyl ester	C109193	C_9_H_18_O_2_	158.2	1074.2	397.938	1.38714	1.22 × 10^3^ ± 84.70	8.69 × 10^2^ ± 91.10	0.006
	Isopentyl butanoate	C106274	C_9_H_18_O_2_	158.2	1060.1	378.974	1.40381	5.11 × 10^2^ ± 45.20	3.71 × 10^2^ ± 57.00	0.017
	Butyl formate	C592847	C_5_H_10_O_2_	102.1	1014	330.632	1.5051	5.57 × 10^2^ ± 77.30	4.87 × 10^2^ ± 1.33×10^²^	0.282
	Ethyl propanoate	C105373	C_5_H_10_O_2_	102.1	1011.2	327.666	1.44949	6.71 ×10^3^ ± 2.54×10^2^	6.11 × 10^3^ ± 2.84×10^2^	0.021
	Butyl propanoate	C590012	C_7_H_14_O_2_	130.2	908.2	257.789	1.27492	5.02 × 10^2^ ± 69.60	8.46 × 10^2^ ± 1.97×10^2^	0.005
	Ethyl acetate	C141786	C_4_H_8_O_2_	88.1	943.6	277.07	1.33409	3.49 × 10^2^ ± 26.40	4.93 × 10^2^ ± 59.90	0.003
	Ethyl butanoate	C105544	C_6_H_12_O_2_	116.2	1014.5	331.173	1.5593	1.10 × 10^3^ ± 1.46×10^2^	7.65 × 10^2^ ± 1.45×10^2^	0.020
	n-Butyl lactate	C34451199	C_7_H_14_O_3_	146.2	1018.6	335.453	1.26872	2.65 × 10^2^ ± 51.10	1.33 × 10^2^ ± 7.96	0.000
	Heptyl acetate	C112061	C_9_H_18_O_2_	158.2	1073.5	396.604	1.448	5.63 × 10^3^ ± 1.87 × 10^3^	4.62 × 10^3^ ± 2.04 × 10^3^	0.811
	Geranyl acetate	C105873	C_12_H_20_O_2_	196.3	1400	1487.894	1.21911	4.14 × 10^2^ ± 59.60	1.83 × 10^2^ ± 32.80	0.000
	*cis*-Rose oxide	C3033236	C_10_H_18_O	154.3	1111.6	473.354	1.38248	3.18 × 10^3^ ± 3.65 × 10^2^	1.68 × 10^3^ ± 2.53 × 10^2^	0.002
Others	Dipropyl disulfide	C629196	C_6_H_14_S_2_	150.3	1098.5	446.929	1.47388	1.23 × 10^3^ ± 2.11 × 10^2^	2.25 × 10^3^ ± 6.31 × 10^2^	0.006
	2,6-Dimethylpyrazine (D)	C108509	C_6_H_8_N_2_	108.1	1327.6	1204.024	1.53744	1.76 × 10^4^ ± 7.42 × 10^2^	1.03 × 10^4^ ± 1.37 × 10^3^	0.004
	2,6-Dimethylpyrazine (M)	C108509	C_6_H_8_N_2_	108.1	1331.2	1218.118	1.14507	3.30 × 10^3^ ± 1.67 × 10^2^	3.09 × 10^3^ ± 1.49 × 10^2^	0.059
	α-Phellandrene	C99832	C_10_H_16_	136.2	1152	554.708	1.67323	4.86 × 10^2^ ± 25.00	1.34 × 10^3^ ± 4.68 × 10^2^	0.000
	4-Methyl-5-vinylthiazole	C1759280	C_6_H_7_NS	125.2	1029.9	347.315	1.53323	4.62 × 10^2^ ± 32.60	1.99 × 10^2^ ± 37.40	0.001
	Decalin	C91178	C_10_H_18_	138.3	1063.2	382.237	1.23101	5.61 × 10^2^ ± 1.44 × 10^2^	1.82 × 10^2^ ± 15.10	0.000
	β-Myrcene	C123353	C_10_H_16_	136.2	977.3	295.442	1.28554	2.45 × 10^3^ ± 2.71 × 10^2^	1.02 × 10^3^ ± 2.30 × 10^2^	0.002
	Ethylpyrazine	C13925003	C_6_H_8_N_2_	108.1	933.6	271.661	1.12623	4.13 × 10^2^ ± 81.50	3.21 × 10^2^ ± 52.40	0.051
	2,3-Diethyl-5-Methylpyrazine	C18138040	C_9_H_14_N_2_	150.2	1154	558.675	1.27281	9.39 × 10^2^ ± 69.70	8.97 × 10^2^ ± 65.40	0.364
	2-Isobutyl-3-Methylpyrazine	C13925069	C_9_H_14_N_2_	150.2	1142.5	535.449	1.30033	1.78 × 10^2^ ± 44.70	1.96 × 10^2^ ± 93.00	0.863
	2-Heptylfuran	C3777717	C_11_H_18_O	166.3	1212.6	763.852	1.40704	3.13 × 10^3^ ± 6.22 × 10^2^	4.40 × 10^3^ ± 2.19 × 10^2^	0.035
	2-Pentylfuran	C3777693	C_9_H_14_O	138.2	1230.8	828.288	1.25257	8.35 × 10^2^ ± 77.00	8.67 × 10^2^ ± 63.50	0.383

* Notes: MW—molecular mass; RI—retention index; Rt—retention time; Dt—drift time. *p*-value was calculated by *t*-test, and the cutoff value was set as below 0.05.

## Data Availability

The data presented in this study are available on request from the corresponding author.
